# An increased potential for organ donors may be found among patients with out-of-hospital cardiac arrest

**DOI:** 10.1186/s13049-022-01037-x

**Published:** 2022-08-17

**Authors:** Mads Anders Rasmussen, Håvard Storsveen Moen, Louise Milling, Sune Munthe, Christina Rosenlund, Frantz Rom Poulsen, Anne Craveiro Brøchner, Søren Mikkelsen

**Affiliations:** 1 The Prehospital Research Unit, Region of Southern Denmark, Odense University Hospital, DK 5000 Odense C, Odense, Denmark; 2 Department of Clinical Research, University of Southern Denmark, Odense, Denmark; 3 Department of Anaesthesiology and Intensive Care Medicine, University Hospital of Southern Denmark, Kolding, Denmark; 4 Department of Regional Health Research, University of Southern Denmark, Odense, Denmark; 5 Department of Neurosurgery, Odense University Hospital, DK 5000 Odense C, Odense, Denmark; 6 Danish Centre for Organ Donation, Aarhus, Denmark

**Keywords:** Organ donation, Prehospital emergency care, Intubation, Level of treatment

## Abstract

**Introduction:**

A prehospital system where obvious futile cases may be terminated prehospitally by physicians may reduce unethical treatment of dying patients. Withholding treatment in futile cases may seem ethically sound but may keep dying patients from becoming organ donors. The objective of this study was to characterise the prehospital patients who underwent organ donation. The aim was to alert prehospital physicians to a potential for an increase in the organ donor pool by considering continued treatment even in some prehospital patients with obvious fatal lesions or illness.

**Methods:**

This is a retrospective register-based study from the Region of Southern Denmark. The prehospital medical records from patients who underwent organ donation after prehospital care from 1st of January 2016–31st of December 2020 were screened for inclusion. The outcome measures were prehospital diagnosis, vital parameters, and critical interventions.

**Results:**

In the five year period, one-hundred-and-fifty-one patients were entered into a donation process in the health region following prehospital care. Sixteen patients were excluded due to limitations in data availability. Of the 135 patients included, 36.3% had a stroke. 36.7% of these patients were intubated prehospitally. 15.6% had subarachnoideal haemorrhage. 66.7% of these were intubated prehospitally. 10.4% suffered from head trauma. 64.3% of these patients were intubated at the scene. In 21.5% of the patients, the prehospitally assigned tentative diagnosis was missing or included a diverse spectrum of medical and surgical emergencies. Twenty-two patients (16.3%) were resuscitated from cardiac arrest. 81.8% were intubated at the scene.

**Conclusion:**

The majority of the patients who became organ donors presented prehospitally with intracranial pathology. However, 30% of the patients that later underwent an organ donation process had other prehospital diagnoses. Among these, one patient in six had out-of-hospital cardiac arrest. Termination of treatment in patients with cardiac arrest is not uncommon in physician-manned prehospital emergency medical systems. An organ donation process cannot be initiated prehospitally but can be shut down if treatment is withheld or terminated. We contend that there is a potential for enlarging the donor pool if the decision processes in out-of-hospital cardiac arrest include considerations concerning future procurement of organ donors.

## Background

Despite an increase in the number of organ donations over the past 10 years, there is a mismatch between the number of organs available for donation and the number of recipients awaiting transplantation in Denmark. At the end of 2021, 389 patients were waiting for transplantation. One-hundred-and five deceased persons entered into an organ donation process [1]. In Denmark, an organ donation process can be initiated if the patient is either declared brain dead or when a patient’s sustained respiratory function is withdrawn resulting in cessation of circulation [2].

New means of procuring organ donors must be investigated. A study from 2017 reported that approximately 30% of potential donors were never admitted to an intensive care unit for donor care but died in the wards [3]. The study suggested that refraining from intubation and mechanical respiratory support were possible reasons for this missed donor potential. International studies suggest that emergency medicine and emergency departments have a role to play in the early identification of potential donors [4]. A report from the Danish Organ Donation Centre suggested that there is a missed donor potential in the prehospital sector [5].

In Denmark, ground-based mobile emergency care units (MECUs) and helicopter-based emergency medical units (HEMS) manned by anesthesiologists are responsible for the prehospitally emergency care for patients suffering acute head trauma and acute intracranial pathologies [6].

The Danish legislation states that a physician is responsible for the decision of whether or not to treat a patient with a prognosis that may seem futile. This prerogative on one hand ensures that patients are not subjected to unethical treatment when there is no reasonable hope for meaningful recovery. Prehospital treatment that from the onset is considered futile for the involved patient may thus be terminated at the scene.

The same prerogative, inadvertently, may allow the prehospital physician to sustain futile treatment after brain death is considered to be imminent. With this study, we aimed to describe the patients that ultimately were entered into a donation process. The aim is thus to alert the prehospital physicians to the potential for increasing the pool of donors by entering considerations concerning future procurement of organ donors into the decision processes in futile cases including patients with out-of-hospital cardiac arrest.

## Methods

### Study setting and population

The population of Denmark consists of approximately 5.8 million inhabitants living in five Health Regions. Each region is responsible for its own provision of health care. The Danish prehospital system is composed of three tiers. This includes the basic unit, an ambulance manned by two emergency medical technicians (EMT) or paramedics (PM). The second tier includes ground-based rapid-response units manned by PMs. The third tier consists of anaesthesiologist-manned units, which can either be ground-based mobile emergency care units (MECUs) or helicopter-based emergency medical services (HEMS) [6]. All emergency calls are handled through a single point of entry for all Danish citizens requiring urgent assistance regarding either fires, police-related problems, or health-related problems. Calls concerning urgent health care problems are transferred to the Emergency Medical Dispatch Centre (EMDC) in each of the five regions. The ensuing handling of all emergency calls is usually carried out by nurses and PMs [7]. The EMDC staff assesses and prioritises the urgency of the health problem and determines the appropriate response.

In the Region of Southern Denmark, approximately 25% of all responses carried out with lights and sirens have a MECU dispatched along with the ambulance [8]. The prehospital anaesthesiologist in the MECU is responsible for the medical treatment. The level of treatment may include intubation and mechanical ventilation, inotropic support, medical treatment of elevated intracranial pressure, antibiotic therapy, and other supportive measures. The details of the findings, the diagnosis, and the prehospital treatment administered are all documented in the prehospital medical record by the anaesthesiologist. The entries into the prehospital medical record are transferred into a national database forming the basis of most of the Danish prehospital research [9]. Each patient is identified via the patient’s Civil Personal Registration Number. This number is a unique identification number assigned to all Danish citizens. By legislation, all citizens with a civil personal registration number are registered in the Danish Civil Registration System. Among others, this registry information about the citizen´s name, place of residence, sex, date of birth, place of birth, and citizenship [10].

### Study design

The study is a retrospective study identifying the prehospital characteristics of all patients included in an organ donation process following prehospital emergency care from the 1^st^ of January 2016 to the 31^st^ of December 2020 in the Region of Southern Denmark.

### Data sources

The data source consisted of prehospital medical records [9]. The organ donors were identified via the Danish Donor Database.

### Inclusion criteria

In the study period, all patients who entered an organ donation process following prehospital emergency care, either by the MECU or by an ambulance on scene were screened for inclusion in this study.

### Exclusion criteria

- According to the approval that was obtained, patients who were transferred from a hospital in another health region were excluded.

### Variables registered

The following patient-related parameters were registered:

- The patient´s age.
- The patient´s sex.
- The first systolic blood pressure.
- The first diastolic blood pressure.
- The first recorded Glasgow Coma Score (GCS).
- The first respiratory frequency measured at the scene.
- The first measured oxygen saturation.
- The tentative diagnosis assigned to the patient by the prehospital anaesthesiologist.

The following system-related parameters were registered:

- The response time.
- The prehospital on-scene time.
- The transport time from the scene to the emergency department.

We also registered variables concerning the medical procedures performed and registered whether any medication was administered. These variables were all recorded as binary parameters. The variables included:

- Tracheal intubation of the patient.
- Intravenous hypertonic saline.
- Other intravenous fluids.
- Oxygen supplement.
- Prehospital opioids.
- Prehospital inotropic medication.
- Other medication.

### Stratification of patients

The patients were stratified according to their prehospitally assigned diagnoses. The strata included stroke, cardiac arrest, subarachnoid haemorrhage (SAH), head trauma, and miscellaneous (convulsions, unspecified intoxication, multi-trauma, acute coronary syndrome, alcohol intoxication, traumatic asphyxia, unspecified injury, pneumonia, observation for suspected disease or condition, unspecified diagnosis, chronic fatigue), and patients with missing prehospital diagnosis. The prehospitally assigned diagnoses were compared with the final diagnoses assigned to the patients following completion of the in-hospital diagnostic procedures.

### Data handling, descriptive analysis and statistical methods

The information about the vital parameters, the system variables, and the prehospital key treatment modalities was extracted manually by the author MAR from the prehospital medical records. [9] Data were entered into Excel files (Microsoft Corporation, Redmond, Washington, USA). Data are presented as proportions or median and quartiles. Proportions are presented with 95% confidence intervals (CI) based on a binomial distribution.

Statistical analyses were performed using STATA 17.0 BE (StataCorp, College Station, Texas, USA). Non-parametric descriptive statistics (medians and quartiles) and non-parametric statistical analyses (Chi-square test and Kruskal–Wallis one-way analysis of variance) were applied, as a normal distribution could not be assured.

### Ethical approval

This study was approved by the Legal Office of the Region of Southern Denmark (J. no. 21/13732). All regulations of the Danish Data Protection Agency were respected. The approval included only data from patients treated in the Region of Southern Denmark.

## Results

In the observation period of five years, a total of 151 patients were entered into an organ donation process at the Odense University Hospital after having received initial prehospital emergency care. Among these patients, 12 were transferred from hospitals in other health regions. The approvals for access to data did only include patients from the Region of Southern Denmark, and patients from other health regions were thus excluded from the study population. Furthermore, four patients were transferred by helicopter and the complete datasets from these patients were not included in the approval from the Legal Office of the Region of Southern Denmark. These four patients were thus excluded.

A total of 135 patients were analysed.

### Demographics

**Fig. 1:**
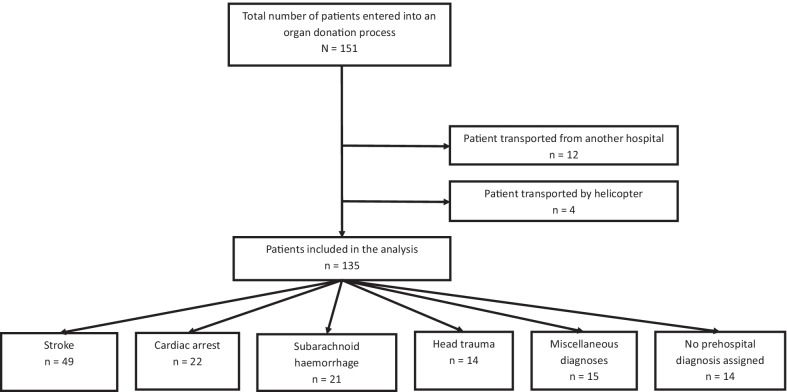
Flowchart of the patients showing inclusions, exclusions, and stratification of patients according to diagnosis

For a flow chart including stratification according to the prehospitally assigned diagnosis, see Fig. 1.

Of the total study population, 73 (54.0%) were women. The median age was 63 years (46 years–73 years). The first recorded median GCS was 4 (3–12). For information about the demographics, vital parameters, and the prehospitally assigned diagnoses, see Table 1.

**Table 1:** Patient demographics and the first measured vital parameters

Prehospital tentative diagnosis	N	Sex (F/M)	Age	*p*-value	Systolic blood pressure	*p*-value	Glasgow Coma Score (Median, Quartiles)	*p*-value	Respiratory frequency (Median, Quartiles)	*p*-value
Stroke	49	26/23	70 (61, 75.5)	0.02	(Median, Quartiles)	< 0.001	10 (3, 14.2)	0.008	20 (19, 22)	< 0.001
Cardiac Arrest	22	8/14	44.5 (32, 63)		175 (150, 195)		3 (3, 3)		0 (0,0)	
Subarachnoideal haemorrhage	21	16/5	71 (62, 76.5)		0 (0, 0)		5 (3.5, 9)		19 (10, 22)	
Head Trauma	14	8/6	49 (34.75,59.75)		157.5 (140, 210)		3 (3, 4)		18 (13, 21.5)	
Miscellaneous diagnoses	15	6/9	45 (36, 55)		142.5 (125, 190)		3.5 (3, 12.5)		20 (19, 24)	
No diagnosis assigned	14	9/5	64.5 (54.5, 77.75)		130 (85, 150)		7 (4, 13.25)		18 (12.25, 24.25)	
Total population	135	73/62	63 (46, 73)		145 (128.75, 188.75)		4 (3, 12)		19 (11, 21.5)	

Miscellaneous diagnoses included: Convulsions, Intoxication, Multi trauma, Acute Coronary Syndrome, Alcohol Intoxication, Asphyxia, Chronic Fatigue, Unspecified Injury, Pneumonia, and Unspecified diagnosis. (Kruskal–Wallis one-way analysis of variance has been applied)

Patients with stroke or subarachnoideal haemorrhage (SAH) were significantly older than the other patients (*p* = 0.02).

Patients with cardiac arrest had significantly lower systolic blood pressure and respiratory frequency, namely zero, at the time of MECU arrival. No clinically important difference in the first measured systolic blood pressure or the first measured respiratory frequency was observed in the other patient groups at the arrival of the MECU at the scene.

Patients with stroke had a higher median GCS than the other patients. This difference was significant (*p* = 0.008). For demographic details, see Table 1.

### System variables

The median response time was six minutes (4–9). The median on-scene time was 26 min (19–34) and the median transport time was 16 min (9–25).

The response time and the transport time were unrelated to the prehospital diagnoses assigned to the patients. However, the on-scene time differed between different diagnosis groups, with patients suffering from cardiac arrest and patients with head trauma requiring the greatest amount of on-scene treatment (*p* < 0.001).

The system variables are shown in Table 2.

**Table 2:** System variables

Prehospital diagnosis	N	Response time (minutes) (Median, Quartiles)	On-scene time (minutes) (Median, Quartiles)	Transport time from the scene to the hospital (minutes) (Median, Quartiles)
Stroke	49	6 (4.5, 9)	22.5 (19, 27.5)	15.5 (9, 26.5)
Cardiac Arrest	22	6 (3.25, 10.5)	39 (29, 45.25)	21.5 (9.5, 32.75)
Subarachnoideal haemorrhage	21	5 (3.5, 7.5)	24(19, 33)	14.5 (8.75, 24)
Head Trauma	14	5 (3, 8.25)	31 (18.5, 42)	14 (5.25, 21)
Miscellaneous	15	8 (4.75, 9.5)	26 (17, 37)	9.5 (5.75, 19)
No diagnosis assigned	14	5 (3, 12.25)	20 (16, 33)	17 (9, 24.5)
Total population	135	6 (4, 9)	26 (19, 34)	16 (9, 25)

Patients assigned a diagnosis of cardiac arrest or head trauma required the largest amount of on-scene time

### Prehospital key treatments

Patients with cardiac arrest were more likely to undergo tracheal intubation and ventilation at the scene compared to patients with other diagnoses. 81.8% (59.7%–94.8%) of these patients were thus intubated at the scene. For details regarding prehospital key treatments, see Table 3.

**Table 3:** Prehospital key treatments

Prehospital diagnosis	N	Prehospital intubation N (% (95%CI))	*p*-value	Hypertonic Saline	*p*-value	Other fluids	*p*-value	Inotropic support	*p*-value
Stroke	49	18 (36.7% (23.4%–51.7%))	0.001	2 (4.1% (0.5%–14.0%)	0.22	9 (18.4% (8.8%–32.0%))	0.80	3 (6.1%(1.3%–16.9%))	< 0.001
Cardiac Arrest	22	18 (81.8% (59.7%–94.8%))		2 (9.1% (1.1%–29.2%))		2 (9.1% (1.1%–29.2%))		18 (81.8% (59.7%–94.8%))	
Subarachnoideal haemorrhage	21	14 (66.7% (43.0%–85.4%))		1 (4.8% (0.1%–23.8%))		3 (14.3% (3.0%–36.3%))		2 (9.5% (1.2%–30.4%))	
Head Trauma	14	9 (64.3% (35.1%–87.2%))		5 (35.7% (12.8%–64.9%))		3 (21.4% (4.7%–50.8%))		5 (35.7% (12.8%–64.9%))	
Miscellaneous	15	8 (53.3% (26.6%–78.7%))		0 (0.0% (0.0%–21.8%))		3 (20.0% (4.3%–48.1%))		3 (20.0% (4.3%–48.1%))	
No diagnosis assigned	14	2 (14.3% (1.8%–42.2%))		0 (0.0% (0.0%–2.3%))		1 (7.1% (0.2%–33.9%))		0 (0.0% (0.0%–2.3%))	
Total population	135	69 (51.1% (42.4%–59.8%))		10 (7.4% (3.6&–13.2%))		21 (15.6% (9.9%–22.8%))		31 (23.0% (16.2%–31.0%))	

Patients with cardiac arrest were significantly more often intubated at the scene and received significantly more inotropic support

37 patients (27.4% (20.1%–35.7%)) were given oxygen supplementation; prehospital opioids were administered in 52 patients (38.5% (30.3%–47.3%)), while 68 patients received medication other than inotropics (50.4% (41.6%–59.1%)).

### Patients presenting with cardiac arrest

Twenty-two patients of the 135 had cardiac arrest upon the arrival of the MECU at the scene. Thirteen of these patients were considered to have cardiac arrest of cardiac origin by the anaesthesiologist in the MECU. Seven patients were considered to have cardiac arrest based on hypoxia, while two patients were considered prehospitally to have cardiac arrest caused by an intracranial catastrophic event.

For details regarding the tentative diagnosis assigned prehospitally and the final diagnosis assigned in-hospitally based on a complete diagnostic workup, see Table 4.

**Table 4:** Diagnosis assigned by (1) The dispatcher over the telephone, (2) The prehospital anaesthesiologists, and (3) The final in-hospital diagnosis

Patient no	Presumed diagnosis assigned by the EMDC dispatcher	Prehospitally assigned diagnosis	Final in-hospital diagnosis
1	Cardiac arrest	Cardiac arrest, hypoxic origin	Cardiac arrest, hypoxic
2	Cardiac arrest	Cardiac arrest, hypoxic origin	Cardiac arrest, hypoxic
3	Cardiac arrest, opioids	Cardiac arrest, hypoxic origin	Cardiac arrest, hypoxic
4	Cardiac arrest, opioids	Cardiac arrest, hypoxic origin	Cardiac arrest, hypoxic
5	Cardiac arrest, hanging	Cardiac arrest, hypoxic origin	Cardiac arrest, hypoxic
6	Cardiac arrest, hanging	Cardiac arrest, hypoxic origin	Cardiac arrest, hypoxic
7	Cardiac arrest, drowning	Cardiac arrest, hypoxic origin	Cardiac arrest, hypoxic
8	Cardiac arrest	Cardiac arrest, cerebral origin	Subarachnoideal haemorrhage
9	Cardiac arrest	Cardiac arrest, cerebral origin	Subarachnoideal haemorrhage
10	Cardiac arrest	Cardiac arrest, cardiac origin	Subarachnoideal haemorrhage
11	Cardiac arrest	Cardiac arrest, cardiac origin	Subarachnoideal haemorrhage
12	Cardiac arrest	Cardiac arrest, cardiac origin	Subarachnoideal haemorrhage
13	Cardiac arrest	Cardiac arrest, cardiac origin	Subarachnoideal haemorrhage
14	Cardiac arrest	Cardiac arrest, cardiac origin	Subarachnoideal haemorrhage
15	Cardiac arrest	Cardiac arrest, cardiac origin	Cardiac arrest, cardiac origin
16	Cardiac arrest	Cardiac arrest, cardiac origin	Cardiac arrest, cardiac origin
17	Cardiac arrest	Cardiac arrest, cardiac origin	Cardiac arrest, cardiac origin
18	Cardiac arrest	Cardiac arrest, cardiac origin	Cardiac arrest, cardiac origin
19	Cardiac arrest	Cardiac arrest, cardiac origin	Cardiac arrest, cardiac origin
20	Cardiac arrest	Cardiac arrest, cardiac origin	Cardiac arrest, cardiac origin
21	Cardiac arrest	Cardiac arrest, cardiac origin	Cardiac arrest, cardiac origin
22	Cardiac arrest	Cardiac arrest, cardiac origin	Cardiac arrest, cardiac origin

## Discussion

While it is common to believe that it is primarily intra-cerebral catastrophes that lead to patients being candidates for organ donation, in this study we found that the patients who later entered into organ donation processes presented themselves prehospitally with many different diagnoses. Thus, 30% of patients who later became organ donors were diagnosed prehospitally with diagnoses other than intracranial haemorrhage, traumatic brain injury, or stroke.

In Denmark, the increased levels of treatment offered prehospitally may be likened to what happens in the initial phase at the emergency department or the intensive care unit. The treatment may thus include tracheal intubation and mechanical ventilation, inotropic support, medical treatment of elevated intracranial pressure, antibiotic therapy, or other supportive measures [8]. The level of treatment offered may be influenced by the perceived prognosis of the patient and the decision to treat the patient or refrain from treatment prehospitally lies solely at the discretion of the attending prehospital physician. In Denmark, it is fully accepted that the physician may terminate or refrain from treatment in cases considered futile already in the prehospital phase. This decision, however, is not only based on medical factors, such as age, comorbidity, or clinical findings. The decision to treat a patient or not has also been shown to depend on many other factors, including non-medical, or intangible factors [11].

The decision to refrain from treatment or withhold treatment may appear ethically sound on the individual level. The possibility that the prehospital physician may terminate the treatment prehospitally implies, however, that the prehospital physician in effect can be considered a gatekeeper for organ donation. A patient in which the treatment may well be considered futile could nonetheless later be considered eligible to enter into an organ donation process.

Previous Danish literature has discussed the potential for increasing the donor pool by increasing the level of the treatment [3]. Considering patients with lethal brain lesions only, one study reported that there is a huge, unrecognised in-hospital donor potential. One of the major points in that particular study was that in patients with lethal brain lesions, withholding tracheal intubation, artificial ventilation, and admission to an intensive care unit will lead to many patients dying without having been considered for the possibility of inclusion in a donation process [3]. The provision of artificial ventilation and admission to an intensive care unit for these patients would undoubtedly increase the number of potential organ donors. This change of focus from the care of a potentially salvageable patient to the care of a patient whose recovery is unlikely with the aim of caring for another patient may, however, be ethically challenging [12].

It has, however, been underscored that actions necessary to facilitate donation in patients who have reached end-of-life care are justified when organ donation is recognised to be consistent with the wishes and interests of a dying patient [13]. These decision processes are not restricted to the intensive care units as potential organ donors may also be identified in the emergency departments. Up to 51% of the time, the decision to initiate end-of-life care starts in the emergency department [14].

### Prehospital presentation of patients that later became organ donors

In our study, the prehospital diagnoses of patients that later underwent organ donation were not restricted to brain injury. Twenty-two patients, who later underwent organ donation, had out-of-hospital cardiac arrest (OHCA). This is a significant finding since patients with cardiac arrest prehospitally otherwise might not routinely be recognised as potential donors. Our findings are in line with other studies that suggest that there is an unrecognised donor potential in out-of-hospital cardiac arrests [15–21]. We concur with these suggestions. In The Region of Southern Denmark, during our study period of five years, approximately 5000 patients suffered from OHCA [22]. In patients without reliable signs of death, prehospital withholding of therapy in OHCA has previously been reported in up to 50% of patients. Of the other 50% who received cardiopulmonary resuscitation, 52% of patients had their treatment terminated at the scene [23]. The termination of treatment in almost 25% of all patients with OHCA without reliable signs of death may point towards a conflict between the ethics surrounding the individual patient and the potential for procuring more organ donors. Although it is unlikely that all of these patients that did not attain a return of spontaneous circulation could have been eligible as organ donors, it seems unlikely that only 22 patients fulfilled the criteria for organ donation. Furthermore, among these 22 patients with OHCA who later became organ donors, seven patients had intracranial haemorrhage which is usually associated with a potential for organ donation. We thus believe that patients with OHCA do represent a potential for increasing the donor pool and it is possible that a higher level of treatment offered to these patients could indeed increase this donor pool. This has been specifically addressed in other countries. A Dutch study investigated the decision-making related to the resuscitation of patients with OHCA. This study found that besides specific medically patient-oriented information (electrocardiographic findings, trauma mechanism), potential organ donation was one of eight themes that influenced the decision-making regarding the level of treatment of prehospital cardiac arrest [24].

In our study, forty-nine patients with a prehospital diagnosis of stroke and 21 patients with SAH became organ donors in the five years. One-third of the patients with stroke and two-thirds of the patients with SAH were intubated at the scene. This left one-third of the patients with SAH unintubated prehospitally. Given the potentially rapid descent in the level of consciousness often seen in patients with SAH, one could speculate if the threshold for intubation and ventilation of these patients should be reconsidered.

In our material, 15 patients were assigned various diagnoses not intuitively associated with the potential for organ donation. This probably reflects the limited diagnostic modalities available prehospitally.

### Strengths of the study

One major strength of the study was that because of the unique Danish Civil Registry [10], which includes all Danish citizens, all patients who underwent organ donation in the Region of Southern Denmark in our study period from 1st of January 2016 to 31st of December 2020 who received emergency prehospital care were screened for inclusion in our study.

Another strength is that all the prehospital medical journals were manually scrutinized and data was manually extracted. This opens up the possibility that any inaccuracies in the medical records could be corrected and clarified.

### Limitations

A major limitation in this study was that the information in the prehospital medical records to some extent was sparse. This may be caused by the very nature of the medical records, where entries in the medical records in effect are supposed to be entered into the record system during treatment or in conjunction with the transfer of the patients to the emergency departments. Most of the patients were, indeed, seriously ill, and the focus from the prehospital physician may in some instances have been primarily on the medical treatment of the patients, not the documentation.

Another limitation in this study is the external validity. The study is also only a single-region study including only patients from the Region of Southern Denmark. Although the emergency medical system in the five Danish health regions principle does not deviate from one another, the density of anaesthesiologist-manned ground-based rapid response units differs somewhat and our results might not reflect the patterns found in the other four health Regions of Denmark to the letter.

## Conclusion

Patients who are included in an organ donation process following a prehospital incident have may present with a multitude of diagnoses and they do not all present with signs of intracranial pathology. One patient in six of those who underwent organ donation presented with cardiac arrest prehospitally. We believe that there is unrealised potential for organ donation within the population of prehospital patients. Prehospital considerations regarding the potential for organ donation should be specifically included in the decision-making when caring for patients having out-of-hospital cardiac arrest and should also be included in decision-making in other patients requiring prehospital life-sustaining therapy. It should be obvious that the process of entering a patient into a transplantation protocol cannot be initiated outside of the hospital. However, some of the procedures necessary to protect the organs while awaiting brain death should be initiated prehospitally. The prehospital care provider does not have the capacity to initiate an organ transplantation process but may well stop it from even becoming a possibility, should necessary life- or organ-sustaining therapies be withheld prehospitally. Prehospital intubation and ventilation may thus be appropriate even in cases that may be considered futile from the start.

We do, however, fully acknowledge that ethical dilemmas may arise, as not all patients whose condition is considered futile prehospitally should receive life-sustaining treatment. There may well be both medical factors and ethical factors that require the prehospital physician to refrain from prehospital treatment or to terminate treatment already initiated at the scene.

## Data Availability

Anonymised data are available on reasonable request.

## Abbreviations

EMDC: Emergency medical dispatch centre
EMT: Emergency Medical Technician
GCS: Glasgow Coma Score
HEMS: Helicopter emergency medical service
MECU: Mobile emergency care unit
OHCA: Out-of-hospital cardiac arrest
PM: Paramedic
SAH: Subarachnoideal haemorrhage
